# Diprotin A TFA Exerts Neurovascular Protection in Ischemic Cerebral Stroke

**DOI:** 10.3389/fnins.2022.861059

**Published:** 2022-05-09

**Authors:** Ming-Yue Zhou, Ya-Jie Zhang, Hong-Mei Ding, Wei-Feng Wu, Wei-Wei Cai, Yan-Qiang Wang, De-Qin Geng

**Affiliations:** ^1^Department of Neurology, The Affiliated Hospital of Xuzhou Medical University, Xuzhou, China; ^2^Department of Neurology, Nanjing Medical University, Nanjing, China; ^3^Department of Neurology, The Third Hospital of Huai'an, Huai'an, China; ^4^Department of Neurology, The Affiliated Hospital of Weifang Medical University, Weifang, China

**Keywords:** ischemic stroke, blood-brain barrier, VE-cadherin, β-catenin, Diprotin A TFA

## Abstract

**Background:**

It has been established that the dipeptidyl peptidase-4 (DPP-4) inhibitor Diprotin A TFA can reduce vascular endothelial (VE)-cadherin disruption by inhibiting the increase in cleaved β-catenin in response to hypoxia, thereby protecting the vascular barrier of human umbilical vein endothelial cells. In this study, we sought to investigate the possible effect of Diprotin A TFA on the VE barrier after cerebral ischemic stroke in mice.

**Methods:**

C57BL/6J mice were divided into five groups, namely, (1) sham, (2) stroke, (3) stroke + dimethyl sulfoxide (DMSO), (4) stroke + Diprotin A TFA, and (5) stroke + Diprotin A TFA + XAV-939. First, the cerebral ischemia model was established by photothrombotic ischemia, followed by intraperitoneal injection with Diprotin A TFA and XAV-939 at doses of 70 μg/kg and 40 mg/kg 30 min once in the morning and once in the evening for 3 days. Immunofluorescence staining and Western blot methods were used to analyze the expression of vascular and blood-brain barrier (BBB)-associated molecular markers in the peri-infarct area.

**Results:**

Compared with the vehicle control group, we found that mice injected with Diprotin A TFA exhibited reduced cerebral infarction volume, increased vascular area and length around the brain injury, increased pericyte and basement membrane coverage, upregulated expression of BBB tight junction proteins, and improved their BBB permeability, whereas the group injected with both drug and inhibitor exhibited significantly aggravated vascular injury and BBB permeability.

**Conclusion:**

Diprotin A TFA can reduce VE-cadherin disruption by inhibiting ischemia-hypoxia-induced β-catenin cleavage to protect blood vessels.

## Introduction

Stroke is well-established as one of the leading causes of death and long-term disability worldwide. During ischemic stroke, additional injury may occur during arterial reperfusion to the tissue following deficient oxygen supply. Such injuries include oxidative stress and disruption of the blood-brain barrier (BBB), followed by intracerebral hemorrhage. The initial ischemic lesions can reportedly enlarge within minutes to hours, whereas the process of activating spontaneous repair mechanisms often lasts weeks to months (Lo, 2008).

Recovery after brain injury is often determined by the residual neural tissue's reorganization. These repair mechanisms include the plasticity of vascular structures and the repair of neurovascular units (Freitas-Andrade et al., 2020). An increasing body of evidence suggests that the BBB is a unique barrier composed of endothelial cells, tight junctions, pericytes, astrocytic terminal foot processes, and basement membrane, essential for regulating the microenvironment (Fischer et al., 1999; Sandoval and Witt, 2008). Interestingly, it has been reported that endothelial permeability is regulated to a certain extent by the dynamic opening and closing of intercellular adherens junctions (AJs). Current evidence suggests that the AJ in endothelial cells is mainly composed of vascular endothelial cadherin (VE-cadherin), an endothelial-specific member of the cadherin family that binds to protein partners through its cytoplasmic domain, including p120, β-catenin, and plakoglobin (Dejana et al., 2008). β-catenin is an essential regulatory protein of the Wnt signaling pathway that can partially bind to E-cadherin to stabilize intercellular adhesion. VE-cadherin and β-catenin play a key role in regulating vascular permeability and integrity (Hashimoto et al., 2017).

A study demonstrated that VE-cadherin exhibited a serrated staining pattern under hypoxic conditions and Diprotin A could alleviate VE-cadherin's disruption by inhibiting β-catenin cleavage in human umbilical vein endothelial cells (HUVEC) (Hashimoto et al., 2017). Moreover, the dipeptidyl peptidase-4 (DPP-4) Inhibitor could exacerbate vascular leakage from the retina by increasing phosphorylation of Src and VE-cadherin in a mouse diabetic retinopathy model (Labat-gest and Tomasi, 2013). From the above literature, it can be concluded that Diprotin A attenuates hypoxia-induced disruption of VE-cadherin by inhibiting β-catenin cleavage in HUVEC, whereas it induces vascular leakage by enhancing the SDF-1α/CXCR4/Src/VE-cadherin signaling pathway. Accordingly, we hypothesized that Diprotin A plays a role in maintaining VE structure integrity after ischemic stroke.

## Materials and Methods

### Animal Grouping

Adult male C57BL/6J mice (weighing 22–30 g) were purchased from the Laboratory Animal Center of Xuzhou Medical University. First, the mice were randomly divided into six groups, namely, (1) control group: (sham operation group); (2) experimental group: 6 h, 1 day, 3 days, 5 days, and 7 days after cerebral infarction. Tissue from the ischemic penumbra area of the cortex (i.e., brain tissue at the junction of ischemic necrotic tissue and normal tissue) was extracted for Western blotting to quantify smooth muscle actin (α-SMA), solute carrier family 16 member 1 recombinant protein (SLC16A1), and Zonula Occludens-1 (ZO-1) protein expression levels. Subsequently, the optimal time point to analyze the expression of the above proteins was selected, then the mice were randomly divided into the following five groups, namely, (1) sham, (2) stroke, (3) stroke + dimethyl sulfoxide (DMSO), (4) stroke + Diprotin A TFA (Diprotin A TFA [DA]: chemical structural analog of Diprotin A), and (5) stroke + Diprotin A TFA + XAV-939 (XAV-939: β-catenin inhibitor). The following experiments were performed using methods such as immunofluorescence staining and Western blotting.

### Cerebral Focal Ischemia

The cerebral ischemia model was induced by photothrombotic ischemia as previously described (Wester et al., 1995; Labat-gest and Tomasi, 2013). Mice were first anesthetized with 10% chloral hydrate intraperitoneally (i.p.) (300 mg/kg in 0.9% saline) and received i.p. injections of 1% Rose Bengal (100 mg/kg in 0.9% saline; Sigma-Aldrich). The head of the mouse was fixed on an animal brain stereotaxic apparatus to ensure that the bregma and λ were in the same horizontal plane (RWD). The scalp was cut open, the anterior fontanel of the skull was exposed, the forelimb representation area of the sensorimotor cortex was identified, and a diaphragmatic pad was placed. The position of the cold light source probe was adjusted to align it with the diaphragm pad, the mouse was given an i.p. injection of 1% Rose Bengal, and the cold light source switched on for irradiation for 12 min (LEICA). At the end of irradiation, the scalp wound was sutured with sutures and the mice were placed in a cage.

### Drug Administration

According to the instructions of the manufacturers, Diprotin A TFA (DA) was diluted in normal saline to a concentration of 20 μg/ml, and the drug was i.p. injected into mice at a dose of 70 μg/kg 30 min before establishing the cerebral ischemia model (Lee et al., 2016). Then, drug injections were given once in the morning and once in the evening 3 days. XAV-939 was diluted to a 3.33 mg/ml concentration in a mixed co-solvent (10% DMSO + 90% corn oil), and XAV-939 was i.p. injected at a dose of 40 mg/kg 30 min before modeling (Wang et al., 2020). The drug was injected once daily for 3 days after the model was established.

### Nissl Staining

Mice were perfused transcardially with phosphate-buffered saline (PBS; 0.1 m phosphate buffer) and 4% paraformaldehyde (PFA) sequentially, and brains were removed and fixed in 4% PFA overnight at 4°C. Then, the murine brains were cut into coronal 40-μm-thick sections using an oscillating microtome (LEICA). Brain sections were sequentially attached to hydrophobic adhesion slides (VICMED), and Nissl staining was performed when the brain slices were dried to transparency. The slides were first placed in 100, 95, and 80% ethanol solutions for 30 s each, then treated with FD Cresyl Violet Solution TM (FD NeoroTechnologies, Columbia, MD, USA) for 5 min, and washed three times with deionized water. The brain slices were sequentially dehydrated in 50, 70, 80, 90, 95, and 100% ethanol solutions for 60 s each, then treated with xylene for 10 min, and finally fixed with neutral resin.

### Infarct Volume Measurement

The brain infarct volume was quantified as infarct volume percentage as previously described (Renolleau et al., 1998; Rousselet et al., 2012; McBride et al., 2015). Cresyl violet-stained brain sections were scanned and imaged using an Epson scanner. The areas of the contralateral hemisphere (*C*_*i*_), ipsilateral hemisphere (*I*_*i*_), and ipsilateral non-ischemic region (*N*_*i*_) were determined using the ImageJ software (NIH), and the infarct volume (%) was calculated as follows:


$$\begin{array}{l} \text{Infarct volume }\left(\text{\%}\right)=\left(\frac{\sum_{i}\left(\left(\frac{I_{i}-N_{i}}{I_{i}}\right)C_{i}\right)}{2\sum_{i}C_{i}}\right)\;x\;100. \end{array}$$

### Evans Blue

The permeability of the BBB was assessed by Evans blue (EB) exosmosis (Goldim et al., 2019; Ahishali and Kaya, 2021). First, 2% EB (3–4 ml/kg; VICMED) was injected into mice *via* the tail vein, and about 1–2 h later, the brains of the mice were perfused and extracted to observe EB extravasation in the cerebral infarction area and assess BBB permeability. After fixing the mouse brain with 4% PFA, the slices were cut with an oscillating microtome to observe EB extravasation under a laser microscope (Olympus).

### Immunofluorescence Staining

Brain sections were blocked with 0.5% TritonX-100 and 10% donkey serum in PBS for 1 h at room temperature, after which the sections were incubated with primary antibodies overnight at 4°C. Primary antibodies included rat anti-CD31 (antiplatelet endothelial cell adhesion molecule-1, labels blood vessels) (Xu et al., 2017) (BD Pharmingen, 553369), rabbit anti-desmin (Cell Signaling, #5332), and rabbit anti-collagen IV (Bio-Rad, 2150-1470). The slides were washed and then incubated with Alexa Fluor 488-conjugated species-appropriate secondary antibody (Vector Labs, DI-1488) and Cy3-conjugated streptavidin (Invitrogen, A10522) for 1 h at room temperature in the dark. Then, the slides were stained with 4′,6-diamidino-2-phenylindole for 5 min in the dark, and the brain slices were washed with PBS, then patched and mounted.

### Western Blotting

First, tissue proteins in the cerebral ischemic penumbra (brain tissue at the junction of ischemic necrotic and normal tissue) were placed in a tissue lysis solution. The homogenates were immediately lysed on ice and centrifuged at 4°C to obtain protein from the supernatant. Then, the protein concentration was measured using a BCA protein assay kit (Beyotime). Equal amounts of protein were loaded, separated by sodium dodecyl sulfate-polyacrylamide gel electrophoresis, and transferred to NC membranes. Then, incubation with primary antibodies and β-tubulin (ProteinTech, 10094-1-AP) was performed overnight at 4°C. Primary antibodies included rabbit anti-α-SMA (Cell Signaling, 19245), rabbit anti-SLC16A1 (NovusBio, NBP1-59656), mouse anti-ZO-1 (Invitrogen, 33-9100), rabbit anti-occludin (Invitrogen, 40-4700), and rabbit anti-PDGFR-β (Cell Signaling, 3169). Then, protein samples were incubated with secondary antibodies for 1 h at room temperature. The target proteins were visualized using a ChemiDoc imaging system (Bio-Rad). For quantification, the density of the blots of interest was normalized to that of tubulin, and optical density was assessed using ImageJ analysis software.

### Image Analysis

Brain angioarchitecture analyses were performed using the open-source “Angiotool” software (National Cancer Institute, USA) (Zudaire et al., 2011). CD31-stained brain sections were used to analyze blood vessel's area and length. The vascular area was defined as the area of the segmented vessel. The vascular length was defined as the sum of the Euclidean distances between all vessel pixels in the image (Gambardella et al., 2012). Desmin- and CD31-positive fluorescent areas were determined to assess pericyte coverage on vessels using the ImageJ area measurement tool. Pericyte coverage was described as the percentage of desmin-positive fluorescent area covering the CD31-positive capillary area (Bell et al., 2010). The basement membrane coverage was defined as the percentage of collagen IV-positive fluorescent area covering the CD31-positive capillary area (Xu et al., 2017).

### Statistical Analysis

All statistical analyses were performed using the GraphPad Prism 8.0 software. For normally distributed measurements, one-way ANOVA followed by Tukey's *post-hoc* test was used for three or more groups. The Kruskal-Wallis test (three or more groups) was used for measurements that were not normally distributed. *p* < 0.05 was considered statistically significant, and data were presented as mean ± SEM from three independent experiments.

## Results

### Changes in Cerebrovascular Sertoli Cell, Endothelial Cell, and BBB Tight Junction Protein After Photothrombotic Stroke

**Figure 1:**
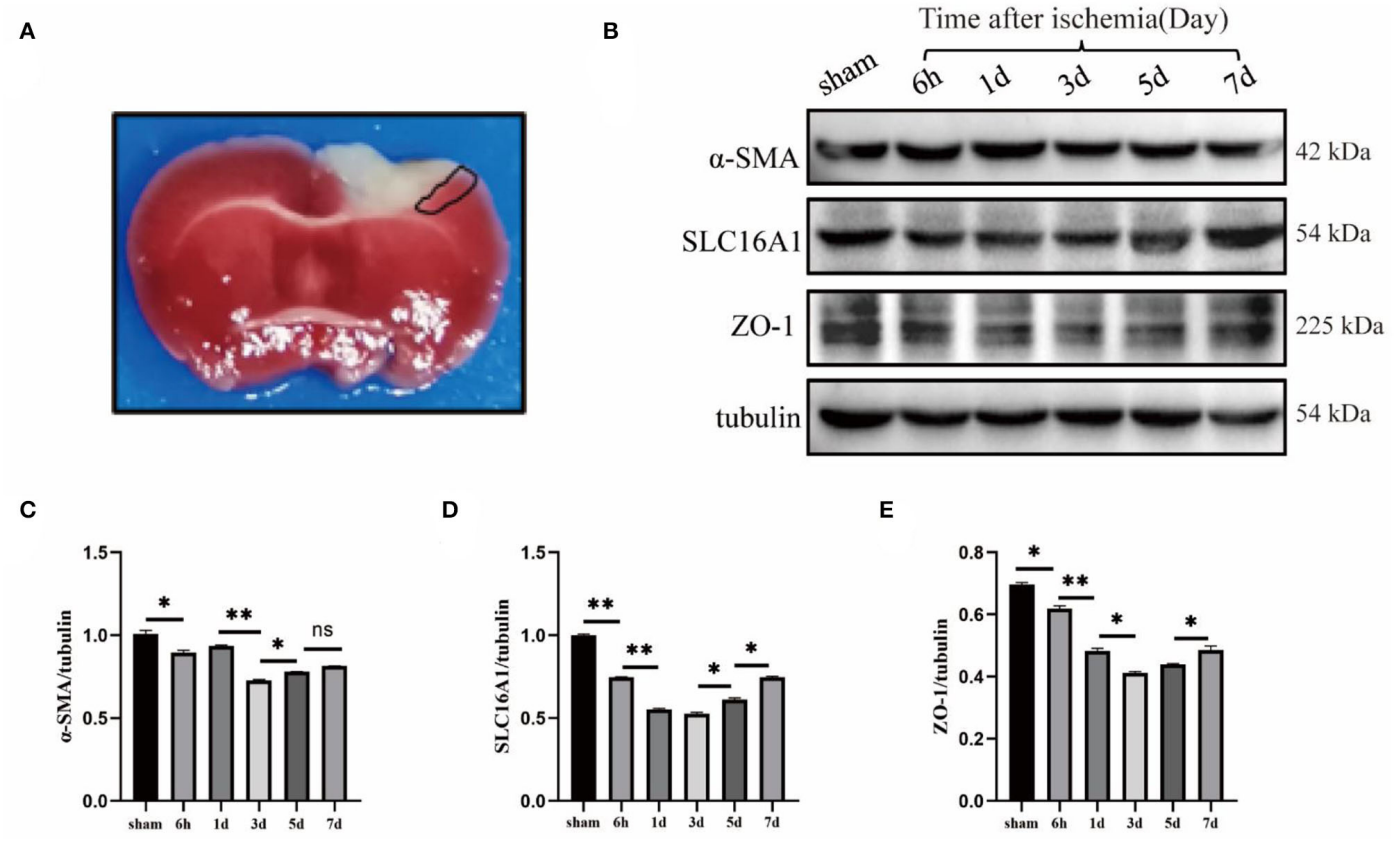
Changes of cerebrovascular Sertoli cell, endothelial cell, and blood-brain barrier tight junction protein after photothrombotic stroke. **(A)** TTC staining image of cerebral ischemia model induced by photothrombosis. The white area of cortex is the cerebral infarction area, and the area surrounded by the black lines is the ischemic penumbra area (i.e., the junction between ischemic necrotic tissue and normal tissue), which is usually used for Western blot (WB) experiment of brain tissue in this area. **(B)** Wildtype mice were used to do sham, 6-h, 1-day, 3-day, 5-day, and 7-day time points of cerebral ischemia model, WB. **(C–E)** The statistical results showed that α-SMA (smooth muscle actin), SLC16A1 (venous-capillary marker) and ZO-1 (Zonula Occludens-1) were all significantly reduced in expression on day 3, which was statistically significant. *n* = 3 in all the experimental groups. Data are represented as mean with SEM. *p < 0.05, **p < 0.01. α-SMA, smooth muscle actin; SLC16A1, solute carrier family 16 member 1 recombinant protein; ZO-1, Zonula Occludens-1.

To understand the expression of pericyte α-SMA (labeled arterioles) (Xu et al., 2017), SLC16A1 (expressed in venous capillary endothelial cells) (Yao et al., 2020), and BBB tight junction protein ZO-1 around ischemic infarcts in the cerebral cortex, Western blot experiments were performed. The mouse brain was intact for 2,3,5-triphenyltetrazolium chloride (TTC) staining after cerebral ischemia (Joshi et al., 2004; Yi et al., 2020), in which the cortex's white area was the cerebral infarction area and the area surrounded by the black line was the ischemic penumbra area; the tissue at this site was harvested for Western blot (Figure 1A). First, wildtype mice were subjected to a sham operation and cerebral ischemia and the ischemic penumbra brain tissues were extracted for Western blot (Figure 1B). The results revealed that the expression levels of α-SMA, SLC16A1, and ZO-1 were significantly reduced on day 3 (Figures 1C–E), indicating that the degree of damage to cerebral vessel structures was the most severe on day 3 after the model was established. The subsequent experiments were performed 3 days after the ischemia model.

### DA Reduces Cerebral Ischemic Damage

**Figure 2:**
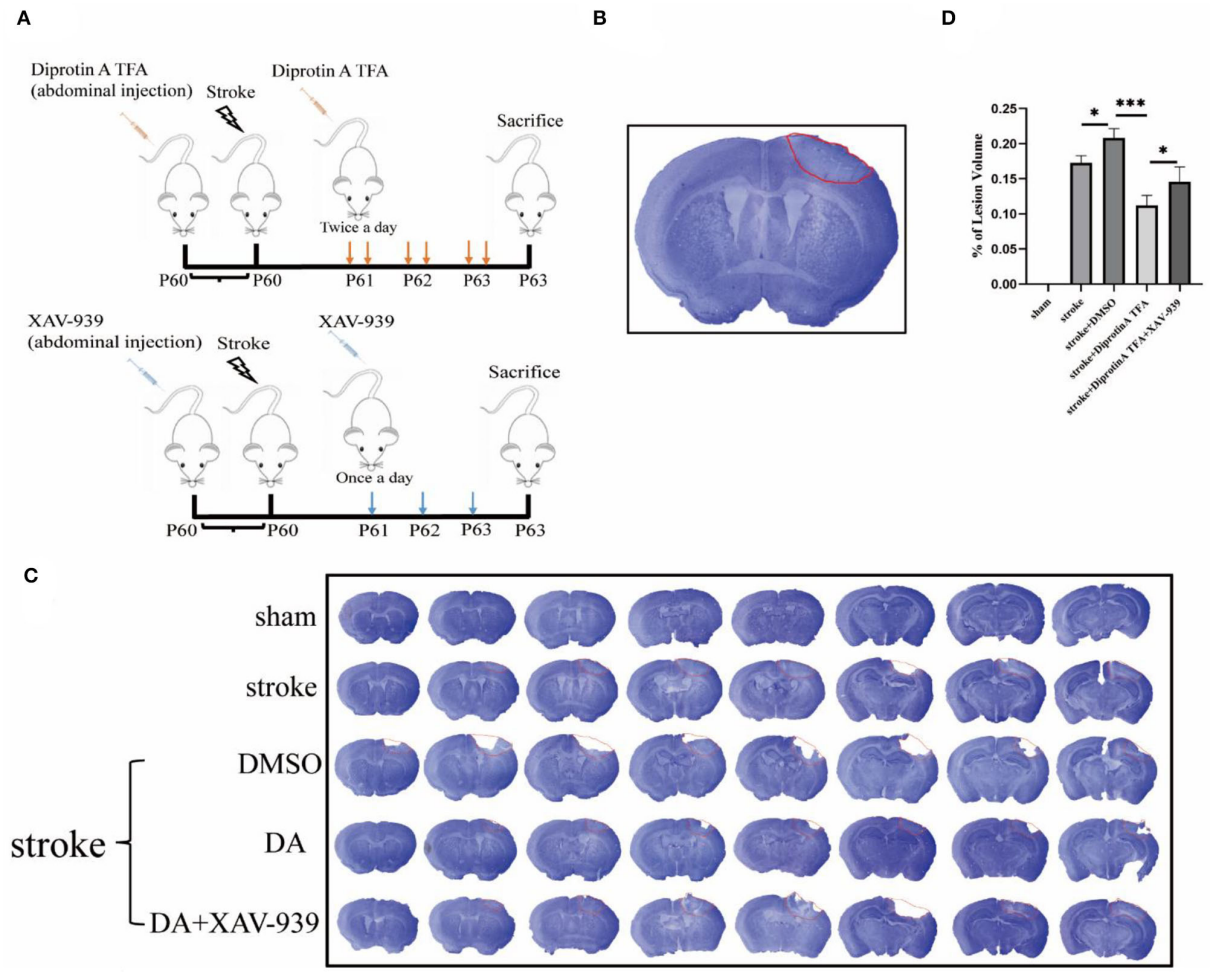
Diprotin A TFA reduces cerebral ischemic damage. **(A)** A simple diagram of the experimental process time of using mice from intraperitoneal injection of drugs to make a model to perfusing the brain or extracting protein. **(B)** A Nissl-stained image of a brain slice, with a section thickness of 40 μm, and the area surrounded by red lines in the upper right part of the brain slice is the infarct area. **(C)** The Nissl staining of five groups of mice after 3 days of photothrombotic cerebral infarction model, The slice thickness is 40 μm. **(D)** Statistical analysis of the cerebral infarction areas of the five groups of mice showed that the cerebral infarction volume of the group injected with DA mice was reduced compared with the vehicle control group. *n* = 3 per group. Data are represented as mean with SEM. *p < 0.05, ***p < 0.001. DA, Diprotin A TFA; XAV-939, β-catenin inhibitor.

First, DA was i.p. injected into mice at 60 days, followed by photothrombosis 30 min later to establish a cerebral ischemia model. DA was injected once in the morning and once in the evening for the next 3 days. XAV-939 was injected as described above, and Nissl staining of brain sections was conducted (Figure 2A). The area surrounded by a red line in the right upper cortex of the brain slice is the infarct area, which is seen to be lighter in color than the surrounding normal tissue (Joshi et al., 2004; Yi et al., 2020), and the cortical infarct areas are all circled with red circles (Figure 2B). The cerebral infarct size was significantly reduced in the DA-treated group and increased in the DA- and XAV-939-treated group (Figure 2C). Moreover, the cerebral infarction volume in the DA-treated group was lower than that in the vehicle control group, the degree of brain injury was mild, and the infarct volume in the DA- and XAV-939-treated group was increased, indicating that XAV-939 exerts an inhibitory effect on the efficacy of DA drug (Figure 2D).

### DA Improves the Permeability of the BBB After Cerebral Ischemic Injury

**Figure 3:**
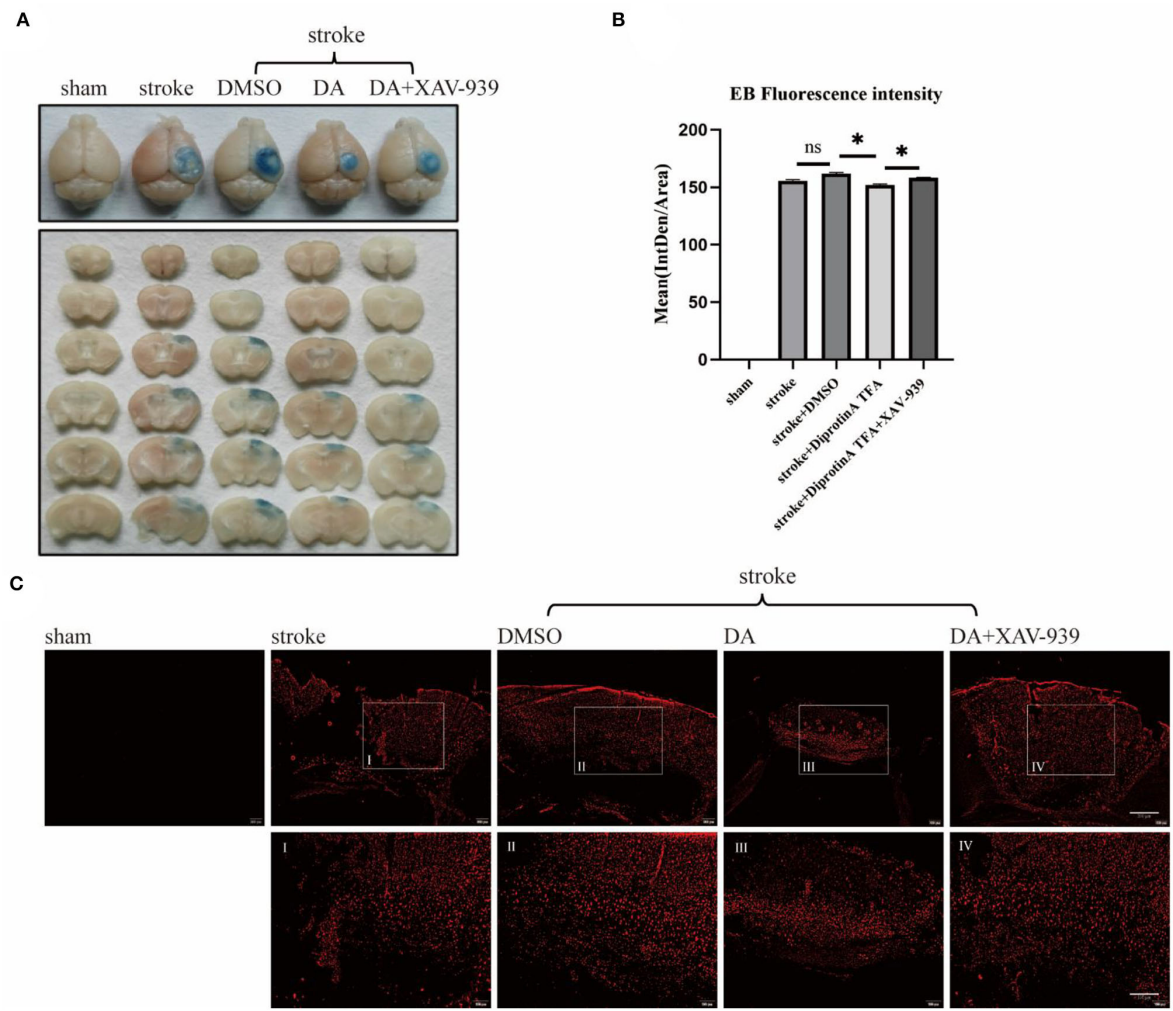
Diprotin A TFA improves the permeability of the BBB after cerebral ischemic injury. **(A)** To inject 2% EB into the tail vein of five groups of mice to observe the extravasation of EB. **(B)** The mean fluorescence intensity of EB extravasation in the cerebral infarction area of the five groups of mice was statistically analyzed. *n* = 3 per group. Data are represented as mean with SEM. *p < 0.05. **(C)** Mice were injected with 2% EB into the tail vein, and the brains were perfused to obtain brain sections for immunofluorescence analysis. The slice thickness is 40 μm. Bar = 200 μm [**(C)**, upper], bar = 100 μ m [**(C)**, bottom]. BBB, blood-brain barrier; EB, Evans blue.

It is well-recognized that EB is an azo dye preparation for assessing capillary permeability since its molecular weight is similar to plasma albumin and its high affinity for plasma albumin in the blood. As plasma albumin does not normally penetrate the BBB, EB bound to plasma albumin cannot stain the BBB if it is intact. Therefore, the permeability of the BBB can be assessed by the extravasation of EB (Goldim et al., 2019). As shown, EB extravasation in the brain-injured area was significantly reduced in DA-treated mice compared with control mice, indicating that BBB permeability was reduced in DA mice, while EB extravasation in the brain-injured area was increased in mice treated with both DA and XAV-939, indicating that XAV-939 could inhibit the drug efficacy of DA to a certain extent (Figure 3A). Statistical analysis of the mean fluorescence intensity of EB extravasation in the cerebral infarction area of the five mice groups showed that EB's mean fluorescence intensity was reduced in the DA-treated group compared with the vehicle control group (Figure 3B). Brain sections obtained after EB injection in mice were observed under a fluorescence microscope, and EB presented red fluorescence only in the cortical infarct area, with red granules as leaky EB (Zhao et al., 2020). Taken together, the above results demonstrated that DA improved BBB permeability (Figure 3C).

### DA Reduces the Degree of Vascular Injury Around Cerebral Infarction

**Figure 4:**
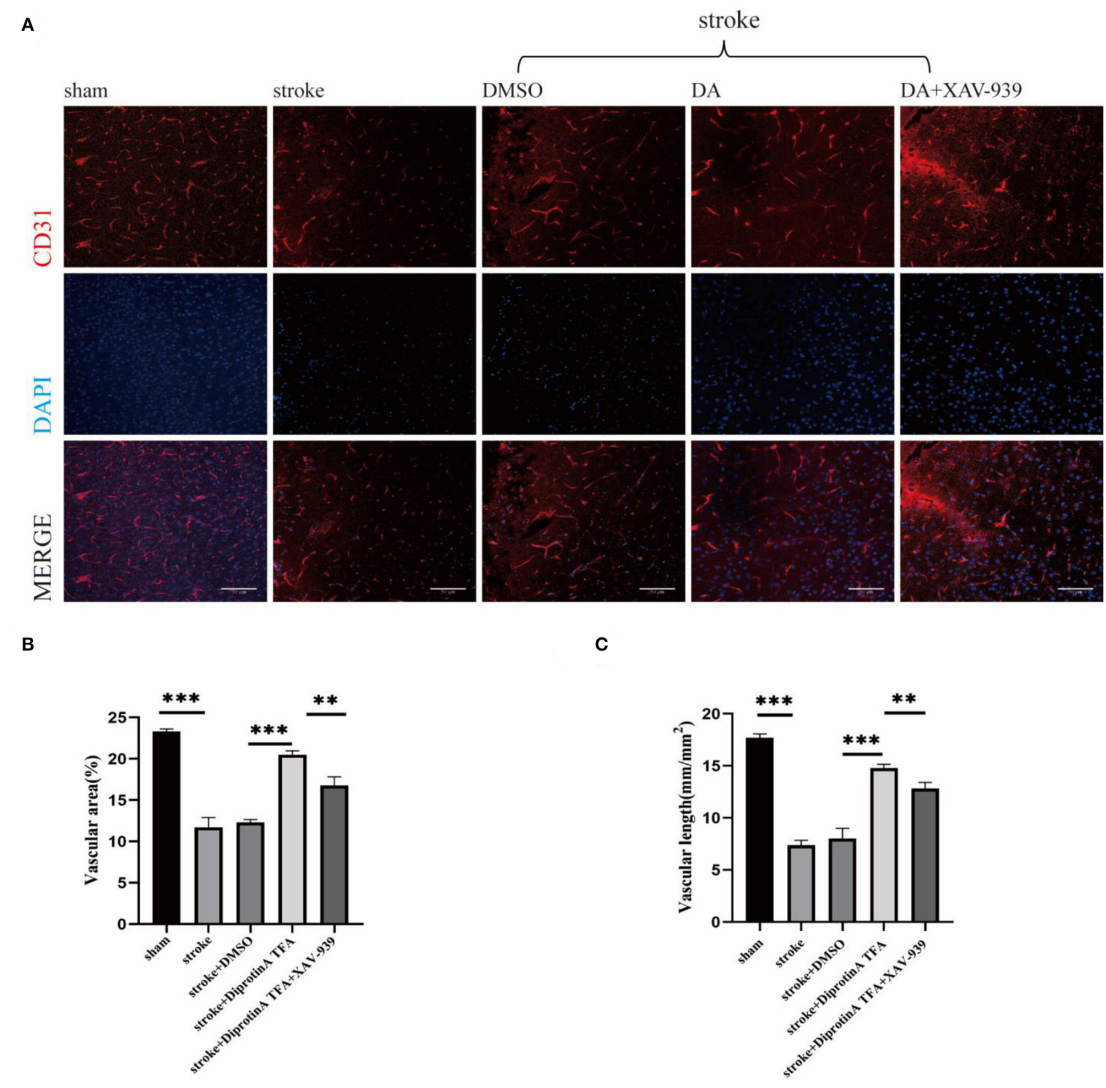
Diprotin A TFA reduces the degree of vascular injury around cerebral infarction. **(A)** There are immunofluorescent staining images of CD31 (red) (antiplatelet endothelial cell adhesion molecule-1), which labels blood vessels. The slice thickness is 40 μm. Bar = 50 μm. **(B)** The statistical plot of vascular area. **(C)** The statistical plot of vascular length. The DA group had a statistically significant increase in the area and length of stained vessels relative to the vehicle control group. *n* = 3 per group. Data are represented as mean with SEM. **p < 0.01, ***p < 0.001.

It is well-established that Diprotin A attenuates hypoxia-induced VE-cadherin destruction by inhibiting β-catenin cleavage in HUVEC (Hashimoto et al., 2017). VE-cadherin is an essential cadherin in VE cells and plays a crucial role in vascular permeability (Dejana et al., 2008). Given that Diprotin A attenuates VE-cadherin destruction after hypoxia, we speculated that DA is essential for the VE structure's integrity. After ischemic brain injury, the vascular density of the surrounding tissue in the cerebral infarction group was reduced compared with the sham group, which was caused by vascular injury after stroke. Accordingly, we explored whether DA induces vascular changes after a cerebral ischemic injury. After conducting immunofluorescence staining for CD31, we found that the vascular density increased in the DA-treated group compared with the vehicle control group (Figure 4A). Statistical analysis of staining images in the five groups showed that vascular area and length were increased in the DA-treated group compared with the control group, but decreased in the group that received both DA and XAV-939, indicating that DA did affect the peripheral vessels of the cerebral infarct, thereby reducing the degree of vascular injury and improving vascular remodeling (Figures 4B,C).

### DA Increases Vascular-Pericyte and Endothelial Basement Membrane Coverage in Peri-Infarct Cortex

**Figure 5:**
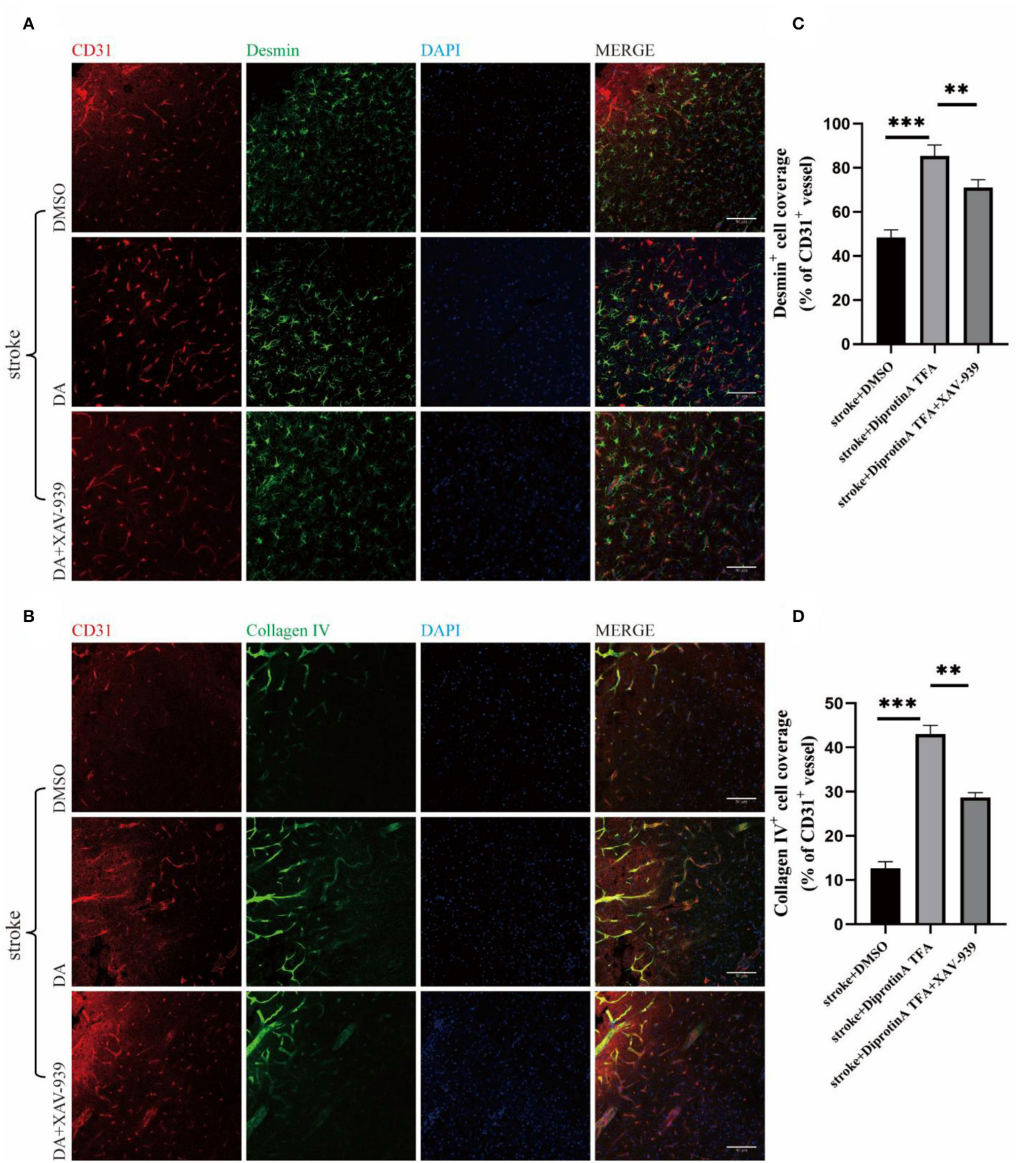
Diprotin A TFA increases vascular-pericyte and endothelial basement membrane coverage in peri-infarct cortex. **(A)** Immunofluorescent staining images of CD31 (red) and desmin (green) (desmin, labeled pericytes) co-staining. The slice thickness is 40 μm. Bar = 50 μm. **(B)** Immunofluorescent staining images of CD31 (red) and collagen IV (green) (type IV collagen, labeled basement membrane) co-staining, with a section thickness of 40 μm. Bar = 50 μm. **(C)** A statistical plot of vascular pericyte coverage. **(D)** A statistical plot of vascular endothelial basement membrane coverage. Compared with the vehicle control group, it can be seen that the coverage rate of stained vascular pericytes in the DA injection group was increased compared with the vehicle control group. Vascular endothelial basement membrane coverage results were the same as above. *n* = 3 per group. Data are represented as mean with SEM. **p < 0.01, ***p < 0.001.

Substantial evidence suggests that the structural integrity of the BBB is mainly composed of endothelial cells, tight junctions, pericytes, astrocyte foot processes, and basement membrane (Kadry et al., 2020). Accordingly, we investigated whether DA could affect BBB integrity. Therefore, brain slices from three groups of mice were subjected to immunofluorescence co-staining for CD31, desmin (labeled pericytes) (Xu et al., 2017), and collagen IV (labeled basement membrane) (Figures 5A,B) (Yao et al., 2020). The results showed that the pericyte coverage of stained vessels in the peri-infarct cortex increased in the DA-treated group compared with the control group and decreased in the DA- and XAV-939-treated group, indicating that DA improved BBB integrity after ischemic stroke (Figure 5C). The statistical analysis results of VE basement membrane coverage were consistent with pericyte coverage, indicating that DA improves BBB integrity after ischemic stroke (Figure 5D).

### DA Regulates the Expression of Tight Junction Protein at the BBB and Pericyte

**Figure 6:**
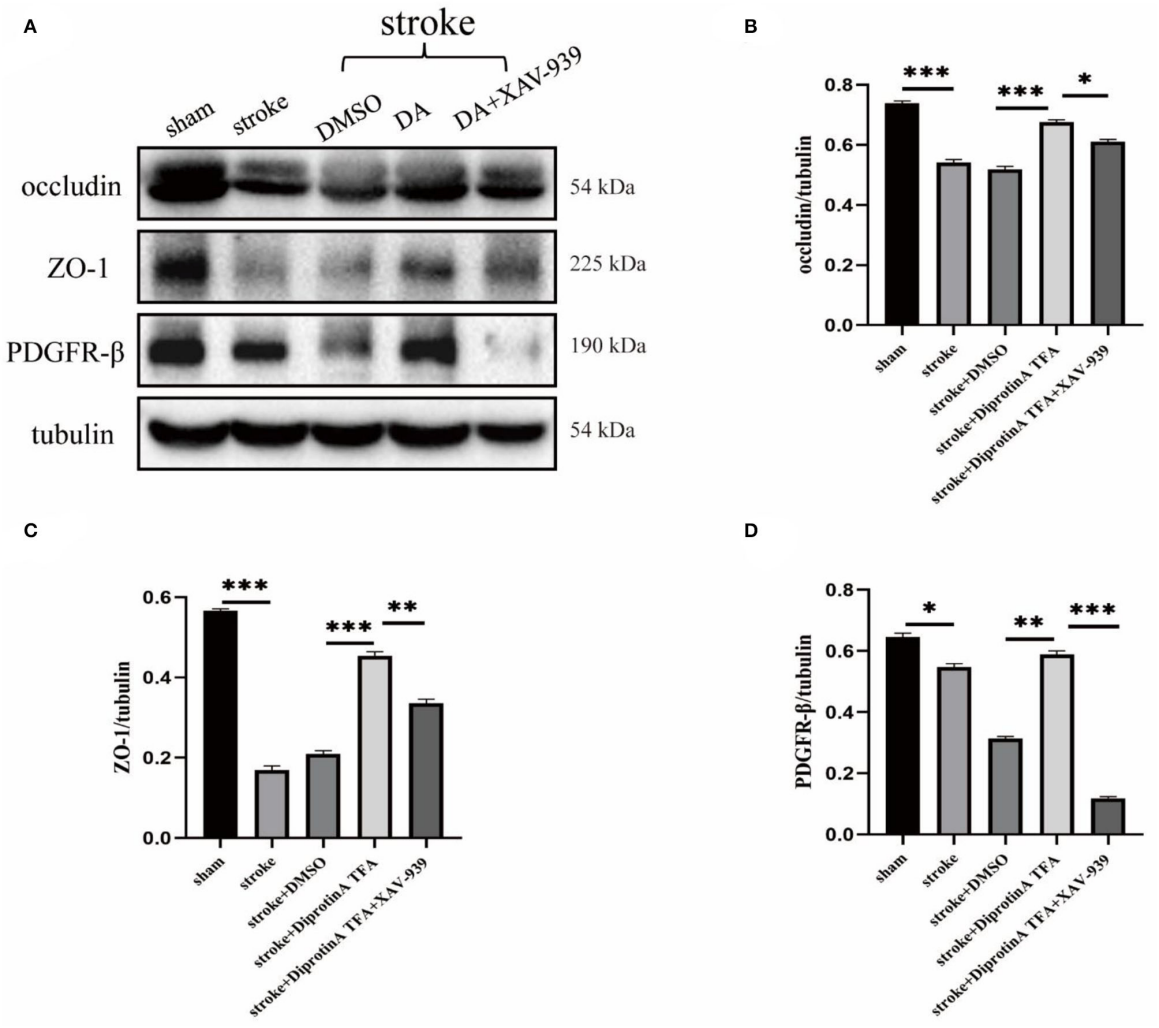
Diprotin A TFA regulates the expression of tight junction protein at the BBB and pericyte. **(A)** Western blot images of BBB-associated tight junction protein (occludin, ZO-1) and pericyte (PDGFR-β) in the penumbra tissue of five groups of mice with cerebral ischemic injury. **(B–D)** The results showed that the expression levels of occludin, ZO-1, and PDGFR-β were upregulated in the group injected with DA compared with the vehicle control group, whereas the expression levels were downregulated in the group injected with DA and β-catenin inhibitor. *n* = 3 per group. Data are represented as mean with SEM. *p < 0.05, **p < 0.01, ***p < 0.001.

Tight junctions and pericytes are reportedly affected when the BBB is damaged (Castro Dias et al., 2019). We subsequently analyzed the expression levels of the associated proteins. Western blotting analysis of BBB-associated tight junction protein (occludin, ZO-1) and pericyte (PDGFR-β) (Bell et al., 2010) was conducted in penumbra tissues of five groups with cerebral ischemic injury (Figure 6A). Statistical analysis showed that occludin and ZO-1 expression levels were increased in the peri-infarct cortex in the DA-treated group compared with the control group, whereas the DA- and XAV-939-treated group exhibited lower levels than in the DA-treated group, and PDGFR-β expression was comparable (Figures 6B–D). These results indicate that DA can upregulate BBB tight junction proteins and pericytes, and can maintain BBB integrity after ischemic stroke. Meanwhile, XAV-939 can downregulate DA and inhibit β-catenin cleavage, attenuating the vasoprotective mechanism of VE-cadherin disruption in the DA- and XAV-939-treated group, thereby downregulating BBB tight junction protein levels and increasing BBB permeability.

## Discussion

In this study, we aimed to explore the effect of DA on the neurovasculature during cerebral ischemia. Importantly, we found that vascular pericytes and the BBB tight junctions were altered following cerebral ischemia. Then, Nissl staining was used to evaluate the cerebral infarction volume, and EB extravasation to assess the BBB structural integrity, vascular area and length, and the pericyte and basement membrane coverage in blood vessels. Findings of this study indicate that DA reduces vascular injury around cerebral infarction, improves BBB integrity and permeability, contributes to vascular remodeling, and is essential for ischemic brain repair.

In recent years, DPP-4 inhibitors have been widely used to treat diabetes (Sun et al., 2020). Interestingly, DPP-4 inhibition improves cardiac function and reduces myocardial ischemia through SDF-1a/CXCR4-mediated STAT3 signaling (Kubota et al., 2016) and exerts an antiapoptotic effect on HUVEC under hypoxic conditions (Nagamine et al., 2017). Accordingly, we hypothesized that DPP-4 inhibitors yield a beneficial effect on cardiovascular and cerebrovascular diseases.

AJs in endothelial cells are mainly composed of VE-cadherin, connected to the AJ proteins p120, β-catenin, and plakoglobin through its cytoplasmic tail (Weis and Nelson, 2006). The importance of VE-cadherin has been emphasized in adult mice for maintaining vascular integrity, given that anti-VE-cadherin antibody administration leads to a dramatic increase in permeability, vascular fragility, and hemorrhage (Corada et al., 1999). Another study that assessed the effect of β-catenin on specific gene inactivation in mouse embryonic endothelial cells suggested its role in vascular permeability and integrity (Cattelino et al., 2003). VE-cadherin and β-catenin are known to play key roles in regulating vascular permeability and integrity irrespective of the mechanism of action (Guo et al., 2008; Rho et al., 2017). Intriguingly, it has been documented that Diprotin A can improve the staining pattern of serrated VE-cadherin by attenuating the increase in cleaved β-catenin levels during hypoxia, thus indicating that Diprotin A protects endothelial AJ from hypoxia (Hashimoto et al., 2017). Therefore, we speculate that Diprotin A exerts a protective effect on the VE barrier during ischemia-hypoxia.

Herein, DA (chemical structural similarity of Diprotin A and DPP-4 inhibitor; Lee et al., 2016) was used to establish a mice model of cerebral ischemia to observe the changes in various parameters. Meanwhile, mice were treated with XAV-939 (β-catenin inhibitor, which can stimulate β-catenin degradation; Huang et al., 2009) and DA to assess the effect of XAV-939 on the pharmacological efficacy of DA. Interestingly, we found that the cerebral infarction volume was significantly reduced in the DA injection group and increased in the DA- and XAV-939-treated group, indicating that DA could improve cerebral ischemic injury, and XAV-939 suppressed the efficacy of DA. Next, immunofluorescence staining using CD31 (Lertkiatmongkol et al., 2016) showed that DA-treated mice had increased vascular area and length in the peri-infarct cortex compared with the control group, and DA alleviated the degree of vascular injury around the cerebral infarction. As shown in Figure 4, vascular density was increased in the DA-treated group compared with the vehicle control group. This effect was induced by vascular injury after an ischemic stroke. It should be borne in mind that the increase in vascular density may be independent of angiogenesis, given that ample evidence substantiates that microvessels on the infarcted side begin to grow 1–2 days after cerebral infarction and peak on day 7 (Morris et al., 2003). However, the optimal experimental time point selected was day 3 after cerebral infarction. The increased vascular density observed in the DA injection group compared with the DMSO injection group (Figure 4) may be attributed to vascular injury. However, the possible role of induction factors of angiogenesis cannot be ruled out. Subsequently, immunofluorescence with CD31 and desmin and collagen IV co-staining were performed. The results showed that the pericyte coverage and endothelial basement membrane coverage of blood vessels in the peri-infarct cortex stained in the DA injection group were increased compared with the control group and decreased in the DA and XAV-939 groups, indicating that DA improved BBB integrity after ischemic stroke. It is widely acknowledged that EB extravasation and tight junction protein expression levels can reflect BBB structure integrity and permeability (Castro Dias et al., 2019; Goldim et al., 2019). Importantly, we demonstrated that DA improves BBB permeability by reducing leakage EB and upregulating tight junction protein content. Our experiments corroborated that DA could alleviate VE-cadherin disruption by inhibiting ischemia-hypoxia-induced β-catenin cleavage, thus exerting a vasoprotective effect. Moreover, the β-catenin inhibitor downregulated this vasoprotective mechanism, which led to the exacerbation of VE-cadherin disruption after hypoxia in the DA and XAV-939 groups, substantiating that DA exerts a protective effect on vascular injury after cerebral ischemia.

## Data Availability Statement

The original contributions presented in the study are included in the article/supplementary material, further inquiries can be directed to the corresponding author/s.

## Ethics Statement

The animal study was reviewed and approved by the Committee on the Ethics of Animal Experiments of Xuzhou Medical University (Xuzhou, China).

## Author Contributions

D-QG and Y-QW formulated the study concept and designed the studies. M-YZ and Y-JZ performed the studies. M-YZ, H-MD, and W-FW executed the experiments and interpreted the results. M-YZ and W-WC assisted in editing the revised manuscript. M-YZ wrote and edited the manuscript. All authors contributed to the article and approved the submitted version.

## Funding

This work was supported by the National Natural Science Foundation of China (No. 81870943) and the Shandong Provincial Nature Fund Joint Special Fund Project (ZR2018LH006).

## Conflict of Interest

The authors declare that the research was conducted in the absence of any commercial or financial relationships that could be construed as a potential conflict of interest.

## Publisher's Note

All claims expressed in this article are solely those of the authors and do not necessarily represent those of their affiliated organizations, or those of the publisher, the editors and the reviewers. Any product that may be evaluated in this article, or claim that may be made by its manufacturer, is not guaranteed or endorsed by the publisher.
